# The effect of organic carbon content on soil compression characteristics

**DOI:** 10.1016/j.still.2021.104975

**Published:** 2021-05

**Authors:** K.N. Suravi, K. Attenborough, S. Taherzadeh, A.J. Macdonald, D.S. Powlson, R.W. Ashton, W.R. Whalley

**Affiliations:** a Rothamsted Research, Harpenden AL5 2JQ, United Kingdom; b The Open University, Milton Keynes MK7 6AA, United Kingdom

**Keywords:** Compressibility, Deviatoric stress, Critical state, Triaxial compression, Effective stress, Soil organic matter (SOM), Soil organic carbon (SOC)

## Abstract

We investigated the effect of soil organic carbon (SOC) on the consolidation behaviour of soil from two long term field experiments at Rothamsted; the Broadbalk Wheat Experiment and Hoosfield Spring Barley. These experiments are located on soil with similar particle size distributions, and include treatments with SOC contents ranging from approximately 1–3.5 g/100 g. Soils taken from plots with contrasting SOC contents were compressed and deformed in a triaxial cell and the normal consolidation and critical state lines were determined. We found that the compression index was independent of SOC, but the void ratio at any given effective stress was highly correlated with organic carbon content. By comparison with uniaxial compression data, the apparent influence of SOC on the compression index is likely to be due to its effect on soil hydraulic properties rather than any intrinsic effects of strength. The plastic limit test appears to be a useful and simple test to allow direct comparison of soil physical behaviour and expected soil density.

## Highlights

• Compression index, from drained triaxial compression, is independent of SOC.
• Reported effects of SOC on compression may be due to soil hydraulic effect.
• Void ratio at any effective stress is strongly correlated with SOC.
• The plastic limit test is a useful test to compare of soil physical behaviour.

## 1 Introduction

**Fig. 1:**
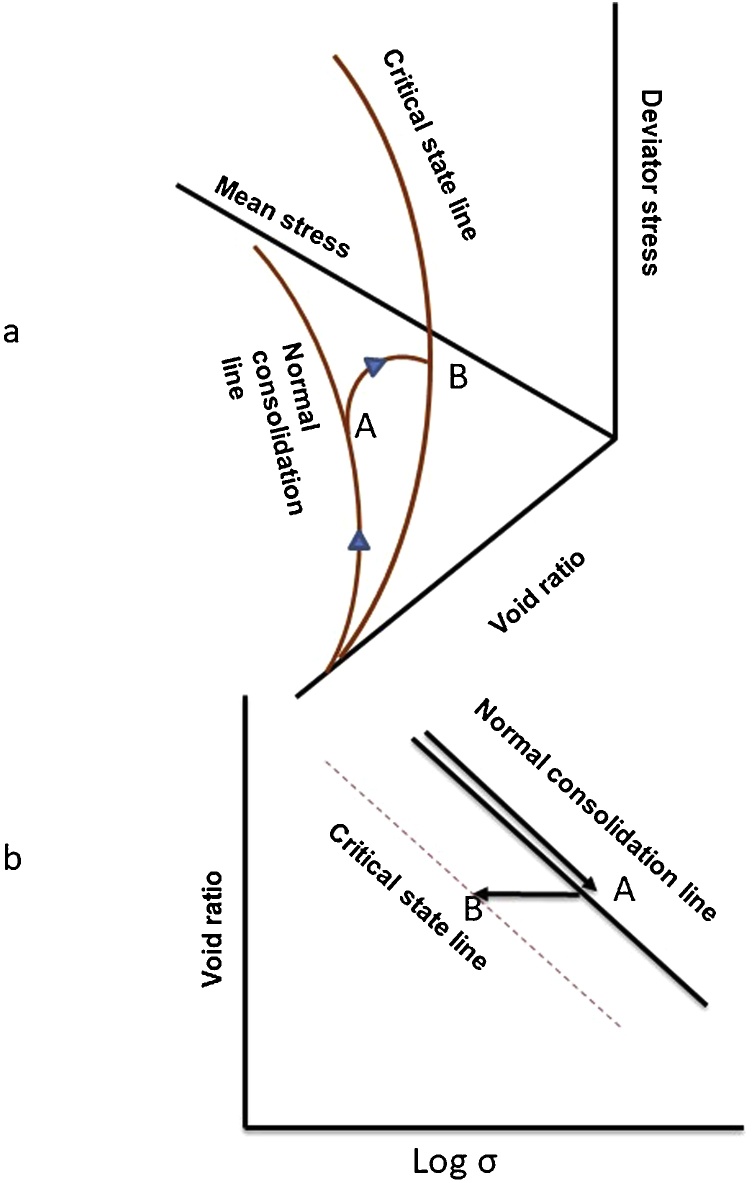
The critical state model of soil deformation. (a) describes consolidation and shear deformation of soil and (b) illustrates normal consolidation line and critical state line on the void ratio vs log mean stress plane. In both cases, route A explains the normal consolidation when soil undergoes to isotopic stress and route B demonstrates shear deformation of undrained soil where the porosity remains constant.

Compaction of an agricultural soil reduces its permeability, modifies biological activity in it and can restrict root growth (Hillel, 1998). During compaction, large pores are destroyed first, and significant changes occur in the pore size distribution. In turn, bulk density increases, reducing the volume of soil macro pores which in turn affects soil physical properties: air permeability, porosity, pore size distribution, hydraulic conductivity and penetration resistance (Whalley et al., 1995). A resistant soil will maintain a high degree of structural function after deformation. To give one example, in a resistant soil, hydraulic conductivity will be maintained at its original value following exposure to an external stress. To make progress in our understanding of how soil structure confers “resistance” we need a framework to allow external stresses to a soil to be related precisely to changes in soil structure. The critical state soil mechanics model is appealing because it allows both consolidation and shear to be related to changes in soil porosity. It allows both elastic and irreversible plastic deformation to be taken into account. Indeed, this approach has been applied to demonstrate the effects of clay content, soil organic carbon (SOC) content and soil water status on the resistance to both isotropic consolidation and shear. The approach has previously been applied to cavity expansion problems around roots (Kirby and Bengough, 2002). When any unconsolidated agricultural soil is loaded, given sufficient external stress, it will move along the normal consolidation line as aggregates fail and reduce in size (see Fig. 1). If the soil is exposed to a shear stress it will lose “structure” by the shear deformation of aggregates until it reaches the critical state line where the soil will be in a similar state to a remoulded or structure-less soil (Fig. 1).

Several studies have described the mechanical behaviour and critical state of agricultural soils (Kirby, 1989, 1998; Wulfsohn et al., 1996; Petersen, 1993; Adams and Wulfsohn, 1997, 1998). The stress needed to deform a soil depends on soil texture and moisture content (Soane et al., 1980; Kirby, 1989; Petersen, 1993).

In contrast to soil moisture content and texture, the effect of soil organic matter (SOM) is not well understood. According to Zhang et al. (2005), SOM due to its complex structure with soil particles acts as a spring against compression and mechanical deformation of soil. Increased SOM content also increases physical stability and mechanical resilience (Griffiths et al., 2005), thus inhibiting destruction of pore structures, and making the soil less susceptible to compaction, by providing mechanical resistance to consolidation and shear deformation (Dexter, 1988; Anderson et al., 1990; Zhang and Hartge, 1995; Zhang et al., 2005). There are too few data that allow an understanding of how soil organic matter, and hence management, affect the critical state parameters. In this study, we have focused on the relationship between compaction and mechanical behaviour of soils in long term agricultural management and the impact of SOM on this relationship. The long-term experiments at Rothamsted provide a source of soils with very different soil organic matter contents, but with a similar soil texture. In this paper we report a series of triaxial and uniaxial tests conducted to understand the relationships between soil organic matter and critical state parameters.

## 2 Materials and methods

### 2.1 Sampling sites

In this study, soil samples were collected from the two oldest continuing long-term experiments at Rothamsted Research, Harpenden, UK. The Broadbalk Wheat Experiment (51° 48′ 32.148′' N, 0° 22′ 15.2112′' W) was established in 1843 and the Hoosfield Spring Barley Experiment (51° 48′ 42.4656′' N, 0° 22′ 32.97′' W) began in 1852 (Macdonald et al., 2018). The Broadbalk soil had 25 % sand, 50 % silt and 25 % clay (Chakraborty et al., 2014) and the Hoosfield soil had a very similar particle size distribution (Glendining et al., 1997). Soil samples (0−23 cm) with a range of SOM contents were collected in May 2016 from 9 plots under continuous winter wheat on section 9 of Broadbalk. This section was selected to minimize the effect of different clay contents (Watts et al., 2006). It is adjacent to a drainage ditch at the east end of the experiment, at the bottom of a slight slope. The treatments sampled included a control plot with no inputs (Nil), a plot given farmyard manure (FYM, 35 t ha^−1^ of fresh manure annually) and plots receiving P and K fertilizers with different levels of nitrogen e.g., PKMg - no nitrogen (BK PKMg); N1 (NPKMg) - 48 kg N ha^-1^ (BK N1) and N6 (NPKMg) - 288 kg N ha^-1^ (BK N6). Soils collected from the Hoosfield Spring Barley Experiment were taken from plots with contrasting fertilizer and manure inputs, including those given inorganic fertilizer (NPKMg) since 1852 (HB 42), continuous farmyard manure (FYM, 35 t ha^-1^ of fresh manure annually) plus N since 1852 (HB 72), FYM + N since 2001 (HB 73) and FYM from 1852 to 1871 plus N since 1968 (HB 71). Initially, N was applied at 48 kg N ha^-1^, but in 1968 a four level N treatment (0, 48, 96 & 144 kg N ha^-1^) was introduced on most plots. Soils were collected by hand using a 2.5 cm diameter gouge auger and then combined to give a single sample for each plot.

### 2.2 Triaxial compression

The triaxial deformation method was used to quantify the normal consolidation curve (NCL, path A, Fig. 1) given by Eq. 1.

(1) $$e=N+\lambda \text{log}\left(\sigma \right)$$

and the critical state line given by Eg. 2 (CSL, path B Fig. 1)

(2) $$\;e=N^{\text{'}}+\lambda^{\text{'}}\text{log}\left(\sigma \right)$$

where e is the void ratio and σ is the effective stress for triaxial data and total stress (i.e. load applied to the solid grains and the water in the pores) for uniaxial data which is described later.

We used a Bishop and Wesley triaxial cell (GDS Instruments, Hook, UK) as described by Chakraborty et al. (2014). The soils were repacked into a split-part mould, 50 mm in diameter (ID) and 100 mm in height, in approximately six layers with an axial pressure of 10 kPa applied by a pneumatic piston. A membrane suction stretcher was used to place the latex membrane around the packed cylinder of soil. The soil sample, surrounded by an impermeable membrane, was placed in position on the pedestal of triaxial apparatus. The triaxial cell and other components were assembled following the placement of the soil sample. The cell was filled with water and pressure / volume controllers connected. First, the sample was saturated at effective stress of 10 kPa, while the cell pressure was increased to 600 kPa and the pore pressure was increased to 590 kPa over a period of 24 h. The saturation process was conducted to ensure all void within the test sample filled with water and any remaining air was forced into solution. Once saturated normal consolidation was applied to the soil sample (NCL, path A Fig. 1) until a mean effective stress of 50 kPa, 150 kPa, 300 kPa or 600 kPa was achieved. During normal consolidation, the cell pressure was maintained at 600 kPa and the pore pressure was reduced over 24 h. At the end of normal consolidation process the soil was sheared, but the volume of the sample was kept constant. The shear deformation was applied by programming the triaxial instrument to decrease the mean stress while increasing the axial stress over a period of up to 8 h. The final weight of the soil specimen after shear test was measured by oven-drying the sample at 105 °C for 48 h.

### 2.3 Uniaxial compression test

Uniaxial compression tests were applied with an Instron 5944 Load frame (UK) running with Instron Bluehill Universal v4.03 software. The soils were packed into plastic rings (inner diameter 46 mm, height 20 mm) with mesh underneath using pneumatic pressure chamber at a low compression pressure of 10 kPa and the soil samples were equilibrated at −10 kPa matric suction on a tension table for 7 days. The soils were then placed on the loading frame (Instron Compression Instrument). The soil samples were compressed with a uniaxial load at a rate of 100 kPa per min up to a maximum force of 1.9 kN. The time (s), vertical displacement (mm), force (kN) and compressive stress (Total stress) (MPa) were recorded. After the compression tests the soil samples were dried at 105 °C for 48 h. Soil N1 (NPKMg) was not compressed due to shortage of sample to run the test.

### 2.4 Determination of soil carbon

Soil carbon (C) was determined by dry combustion using a LECO TruMac analyser (LECO, Michigan, USA). Soil samples were air-dried and finely milled to make a homogenous sample. About 0.4 g finely milled soils was accurately weighed into ceramic boats and used to determine C content. SOC was determined by subtracting the amount of inorganic C (though this was negligibly small) from total C.

### 2.5 Consistency limits measurement

Plastic and liquid limits of both soils (Broadbalk and Hoosfield) were measured following the British standard (BS 1377-2: 1990). For both measurements soil samples were air dried and ground finely and passed through 425-micron sieve. About 400 g of finely ground soil were taken for the measurements and thoroughly mixed with distilled water on a glass plate and placed in the airtight plastic bag for a suitable maturing time.

### 2.6 Plastic limit measurement

The hand rolling method has been followed, and as described by Keller and Dexter (2012), to measure plastic limits of soil samples. About 20 g from the soil paste prepared were taken to measure plastic limit of soils.

### 2.7 Liquid limit measurement

The liquid limit of soil is the absolute moisture content at which soil passes from a liquid state to a plastic state. The liquid limit of soil samples were measured with the cone penetrometer method. Because this method is very easy to conduct and produce more reproducible data. In this method, a cone of stainless steel is used which is approximately 35 mm long with polished surface and an angle of 30°. About 300 g from the prepared soil paste was taken for liquid limit measurement. Water contents of soils were determined by oven drying the soils.

### 2.8 Statistical analysis

All statistical analyses of collected data were carried out using GenStat V17. Data were analysed with ANOVA and grouped regression analysis. Deformation testing in the triaxial cell was randomised according to both the soil treatment and the final pressure during normal consolidation. The testing procedures of all soil samples were then conducted according to a prearranged test sequence of 40 independent tests.

## 3 Results

### 3.1 Soil organic carbon and consistency

The soil organic carbon contents and consistency limits are shown in Table 1. Soil organic carbon content explained 98 percent of the variance in plastic limit, as is widely reported (Keller and Dexter, 2012).

**Table 1:** Selected properties of soils from Broadbalk and Hoosfield.

Site	Soil treatment	SOC, g/100g		Plastic limit, g/100g	SE	Liquid limit, g/100g	SE
Broadbalk (BK)	FYM	2.96	±0.016	26	±0.387	43	±2.17
	Nil	0.85		20		36	
	PKMg	0.91		20		37	
	N1(P)KMg	1.08		21		40	
	N6(P)KMg	1.26		21		35	
Hoosfield (HB)	FYM since 1852	3.65	±0.001	27	±0.549	45	±3.04
	FYM since 2001	2.18		23		37	
	FYM from 1852−71	1.45		22		40	
	N(P)K(Mg)	0.99		20		36	

### 3.2 Consolidation characteristics

**Fig. 2:**
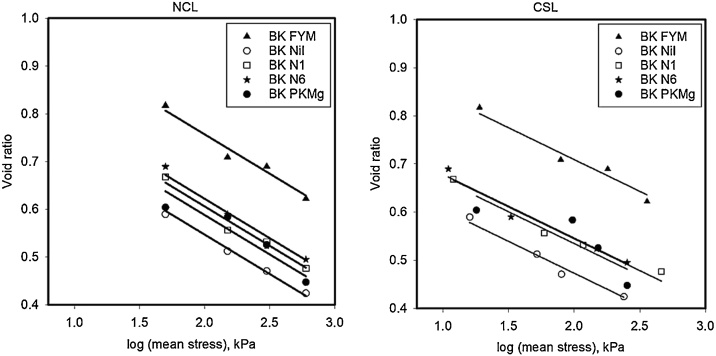
Normal consolidation (P < 0.001; s.e.d = 0.01; Var. = 0.968) and Critical state lines (P < 0.001; s.e.d = 0.02; Var. = 0.952) for Broadbalk soils shows the similar slopes with different intercepts. The NCL data were obtained from drained triaxial testing with a Bishop and Wesley cell. The CSL data were obtained following shear deformation at a constant volume.

The initial density, prior to triaxial testing depended on SOC and ranged from approximately 1.0–1.2 g cm^−3^ for samples with the highest to the lowest SOC, respectively. Following triaxial testing, the void ratio of different soils was plotted against logarithm of mean stress for the NCL and CSL conditions in Figs. 2 (Broadbalk) and 3 (Hoosfield). Grouped regression showed that the data could be described with a set of parallel curves with different intercepts when analysed with Genstat®. (Table 2). Different slopes were needed to explain the effects of soil location (i.e. Broadbalk or Hoosfield) as well as for the NCL and CSL conditions, although all of the slopes (for both NCL and CSL) were similar in value. Among the samples from Broadbalk, the FYM sample had the highest porosity (void ratio = 0.97 at critical state) whereas the smallest porosity (void ratio = 0.73 at critical state) was observed from soil samples from the Nil (control) plot. Similarly, on Hoosfield, soil from the plot given FYM since 1852 (highest SOM content) showed the highest porosity (void ratio = 1.2 at critical state) (Fig. 4, Table 3).

**Fig. 3:**
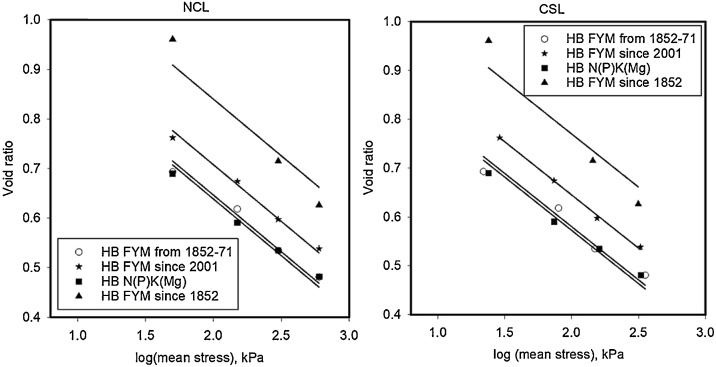
Normal consolidation (P < 0.001; s.e.d = 0.008; Var. = 0.995) and Critical state lines (P < 0.001; s.e.d = 0.01; Var. = 0.992) for Hoosfield soils. The NCL data were obtained from drained triaxial testing with a Bishop and Wesley cell. The CSL data were obtained following shear deformation at a constant volume.

**Table 2:** Parameters of the NCL and CSL estimated from linear regression while grouped regression explained 90 percent of variance in void ratio.

Site	Soil treatment	Normal consolidation line (NCL)		Critical state line (CSL)	
		Slope, λ (±SE)	Intercept, N (±SE)	Slope, λ’ (±SE)	Intercept, N’ (±SE)
Broadbalk (BK)	FYM	−0.165 (±0.010)	1.087 (±0.024)	−0.132 (±0.010)	0.973 (±0.023)
	Nil		0.877 (±0.024)		0.737 (±0.021)
	PKMg		0.918 (±0.024)		0.799 (±0.023)
	N1(P)KMg		0.936 (±0.024)		0.809 (±0.022)
	N6(P)KMg		0.952 (±0.024)		0.810 (±0.021)
Hoosfield (HB)	FYM since 1852 (72)	−0.228 (±0.016)	1.297 (±0.039)	−0.217 (±0.016)	1.206 (±0.037)
	FYM since 2001 (73)		1.165 (±0.038)		1.081 (±0.036)
	FYM from 1852−71 (71)		1.103 (±0.038)		1.016 (±0.036)
	N(P)K(Mg) (42)		1.096 (±0.038)		1.008 (±0.036)

**Fig. 4:**
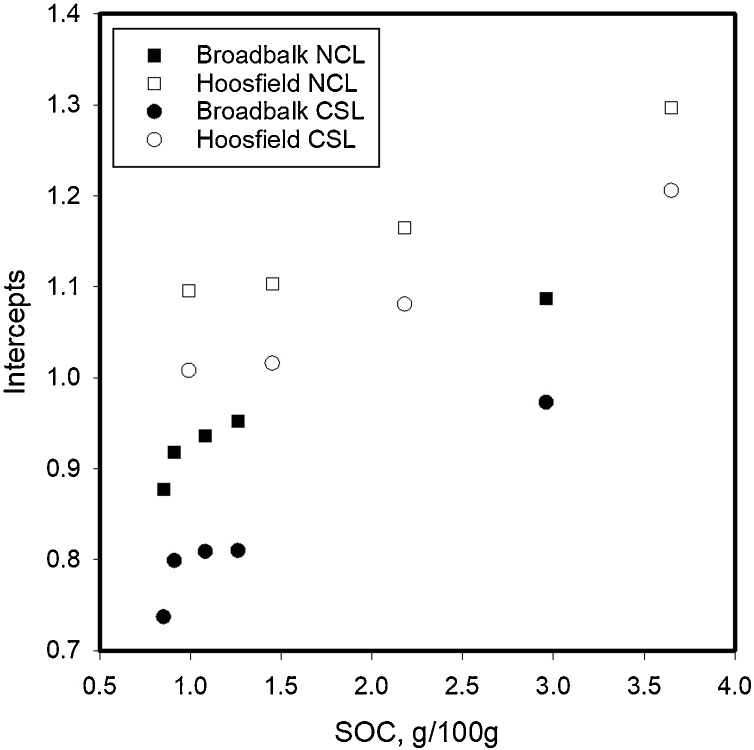
Intercepts of NCL and CSL obtained from fits to the data in Fig. 2, Fig. 3 plotted against SOC.

**Table 3:** Intercepts vs OC (Fig. 4).

Source	Soil	Slope (±SE)	Intercept (±SE)	F probability (Grouped regression)
NCL	Broadbalk	1.074 (±0.03)	0.7984 (±0.05)	0.002
	Hoosfield	1.837 (±0.04)	0.7869 (±0.08)	
CSL	Broadbalk	1.139 (±0.1)	0.6622 (±0.03)	<0.001
	Hoosfield	1.737 (±0.2)	0.7233 (±0.04)	

**Fig. 5:**
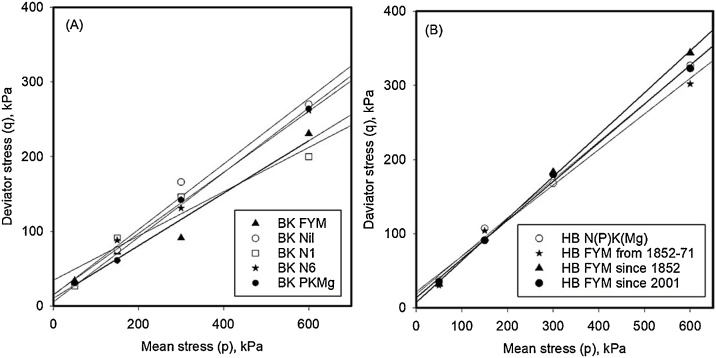
The projection of consolidation behaviour of soils from Broadbalk (A) and Hoosfield (B) on stress (p-q) plane (see Fig. 1).

The straight lines fitted to plots of mean effective stress against the deviator stress (p–q) diagram are shown in Fig. 5. While the projection of critical state lines for both soils did not pass through the origin, the slopes ranging from 0.3 to 0.6 are similar to those reported by O’Sullivan and Robertson (1996) for wet soil (0.1 to 0.6). In the stress plane, Hoosfield soil is stiffer than Broadbalk soil.

### 3.3 Uniaxial compression of soils

In the results from the uniaxial compression tests N2(NPKMg) - 96 kg N per ha (BK N2) was used instead of N1(NPKMg). The compression indices of soils from the uniaxial tests are shown in Table 4. These data suggest that the compression index depends on SOC in the more rapid uniaxial compression test when total stress is used, but not in the slower drained triaxial test when void ratio is plotted against effective stress.

**Table 4:** Compression indices of sampled soils with statistical analysis (regression with their respective SOC content) derived from uniaxial compression test.

Site	Soil treatment	SOC, g/100g	Compression index	Statistics	
				F probability	s.e.
Broadbalk (BK)	FYM	2.959	0.391	0.03	±0.047
	Nil	0.847	0.288		
	PKMg	0.915	0.288		
	N2(P)KMg	1.156	0.248		
	N6(P)KMg	1.265	0.351		
Hoosfield (HB)	FYM since 1852	3.650	0.343		
	FYM since 2001	2.181	0.327		
	FYM from 1852−71	1.454	0.248		
	N(P)K(Mg)	0.991	0.259		

## 4 Discussion

**Fig. 6:**
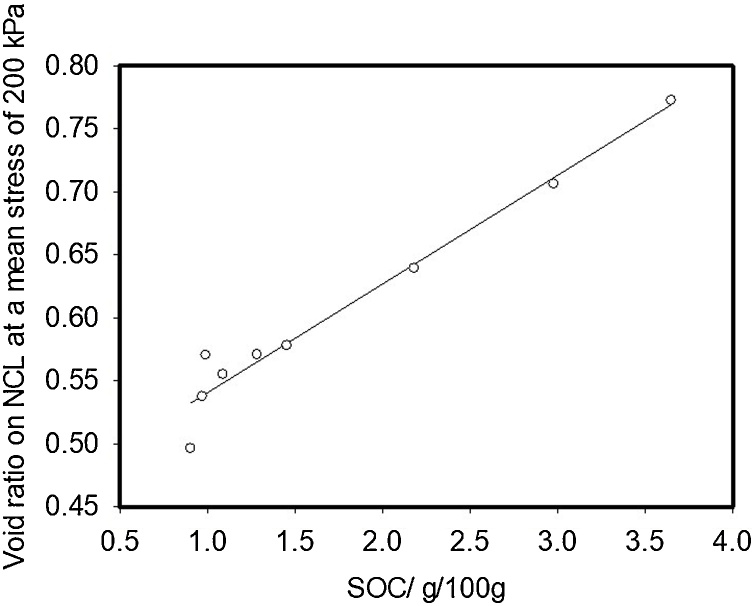
The void ratio of soil in normal consolidation an effective stress of 200 kPa, obtained by interpolation with the curves shown in Fig. 2, Fig. 3, plotted against soil organic carbon.

In this study we compared the compression characteristics, obtained from drained tests, of soils with a similar particle size distribution, but with a range of SOCs. Following compression soil samples were sheared at a constant volume. We found that, for Broadbalk and Hoosfield soils, the slope of the compression characteristic, or the compression index from the slow triaxial compression, depended on the source of the soil (i.e. whether it was from the Broadbalk or Hoosfield experimental plots) but not on the amount of organic carbon in the soil (Table 2). The compression indices for the NCL and CSL for Hoosfield and Broadbalk were similar, although O’Sullivan et al. (1994) found that CSL lines showed greater slope than NCL. The change in void ratio due to an increment in effective stress can be written as

(3) $$de=\text{Λ}\frac{d\sigma }{\sigma }$$

where Λ is a lumped parameter (plastic compression index) that takes account of total and elastic strain during compression (McDowell et al., 1996). Our results show that for a given soil texture, Λ is constant and does not depend on organic matter content. According to the analysis of McDowell et al. (1996), if there is a low variability in particle strength, a similar slope in the compression characteristic implies a similar fractal dimension. In an agricultural context, compression pressures of no more than 1 MPa are small compared with up to 10 MPa or greater which are frequently used in civil engineering studies. For 0.5 mm quartz grains to fracture, approximately 50 MPa will be needed (Cheng et al., 2001; De Bono and McDowell, 2018). Thus, in our experiments a particle can be conceptualised as a transient collection of primary particles (or aggregates) held together by organic matter, which break into smaller units with increasing pressure. McDowell et al. (1996) further explain that, $\text{Λ}\;\text{α}\;{\text{Γ}}^{1-m(D-1)/4}$, where Γ is the surface free energy, *m* is the Weibull modulus and *D* is the fractal dimension. Our data imply that all these parameters are independent of soil organic matter. However, the void ratio of soil does depend on organic matter content. Fig. 6 shows that the void ratio of soil in normal consolidation at an effective stress of 200 kPa, obtained by interpolation from the fitted curves, is closely correlated with the soil organic carbon content. Although the void ratio depends closely on organic matter, at a given effective stress, an additional increment in effective stress will give the same reduction in void ratio in all the soils we tested according to Eqs. 3, albeit that Λ will depend on the soil type (Broadbalk or Hoosfield). McDowell (2005) showed the fractal compression was consistent with a compression characteristic written in the form

(4) $$\text{ln}\text{e}=\text{ln}{\text{e}}_{\text{y}}-\frac{1}{2\text{b}}\text{l}\text{n}\frac{\text{σ}}{{\text{σ}}_{\text{y}}}$$

The parameter b deﬁnes the rate at which average particle strength increases with decreasing size and, given the slope of the compression characteristic, is independent of organic matter, the same is true for b (Table 5) which was not correlated with soil organic matter. It is implicit that irrespective of the void ratio similar patterns of aggregate failure occur. This suggests that the structures that confer strength to aggregates, thereby resistance to compression, are similar at all size scales.

**Table 5:** Parameters from ln $e_{y}$ vs lnσ relationship.

Site	Soil treatment	Slope, 1/2b	Estimated lne_y_	Calculated lne_y_
Broadbalk (BK)	FYM	0.105	−0.048	−0.184
	Nil	0.131	−0.176	−0.346
	PKMg	0.132	−0.155	−0.308
	N1(P)KMg	0.137	−0.127	−0.298
	N6(P)KMg	0.118	−0.109	−0.289
Hoosfield (HB)	FYM since 1852	0.171	0.021	−0.231
	FYM since 2001	0.141	−0.082	−0.290
	FYM from 1852−71	0.150	−0.107	−0.316
	N(P)K(Mg)	0.144	−0.103	−0.291

Zhang and Hartge (1995) reported inconsistent accounts of the effects of soil organic carbon on soil compression index. Some recent data are summarised in Table 6, show that compression index increases with clay content and organic matter content. However, soil organic carbon and clay content are correlated, as can be seen in the data of Gregory et al. (2006) in Table 6. Our results from more rapid uniaxial compression tests of initially wet soil, may reflect differences in hydraulic conductivity rather than an intrinsic mechanical property. In our study we did not find any effect of soil carbon content on the compression index, when obtained from a slow drained triaxial test. 'However, void ratio at any particular effective stress was strongly correlated with soil carbon content (Fig. 6). In uniaxial compression tests, Keller et al. (2011) also found that the compression index was independent of organic matter, but that it was dependent on the initial void ratio, which, for a given soil is not entirely consistent with Fig. 1. However, Keller et al. (2011) used a uniaxial test where drainage of initially wet soils (at ψ =−100 hPa) was needed for consolidation to occur. In uniaxial compression tests Gregory et al. (2006) also found the compression index depended on initial void ratio, for the same soils. It is possible that soils with a higher void ratio were more compactable because drainage is closely correlated with void ratio (Whalley et al., 2012). It is also likely that the pore pressures during uniaxial compression by differ between the soils with different SOC (e.g. Huan et al., 2021). Other studies have also used similar compression tests (Arthur et al., 2012) and found a positive relationship between initial void ratio and compression index. Interestingly, Arthur et al. (2012) compared three agronomic treatments which had different soil organic carbon content, but similar textures. The different agronomic treatments, mixed forage cropping, mixed cash cropping and cereal cash cropping had organic carbon contents of 2.1, 1.4 and 1.0 g/100 g, respectively. The treatment with higher organic matter had the smaller compression index. They concluded that soils with higher soil organic matter content were better able to resist changes in bulk density.

**Table 6:** Compression index of different soils from Bedfordshire, UK by axial compression with other selected properties (Gregory et al., 2006).

Soil	Location	Sand (0.06-2 mm), g/100g	Silt (0.002-0.06 mm), g/100g	Clay (<0.002 mm), g/100g	SOC, g/100g	Compression index (estimated by Gompertz model)	
						0–100 kPa	0–200 kPa
Calcareous pelosol	Boot Field I	9.8	14.3	75.8	4.4	1.411	1.210
	SRC	39.4	19.5	41.2	3.6	1.191	1.001
	Clover Hill	9.8	19.2	71.0	5.1	1.328	0.999
	Long Shot	31.9	15.7	52.4	3.8	1.221	0.809
Brown sand	Cashmore II	48.9	14.6	36.5	3.2	0.837	0.697
	East Drive	69.7	13.9	16.4	1.4	0.629	0.528
	Burton’s Gate	68.0	17.9	14.2	1.7	0.606	0.547

**Fig. 7:**
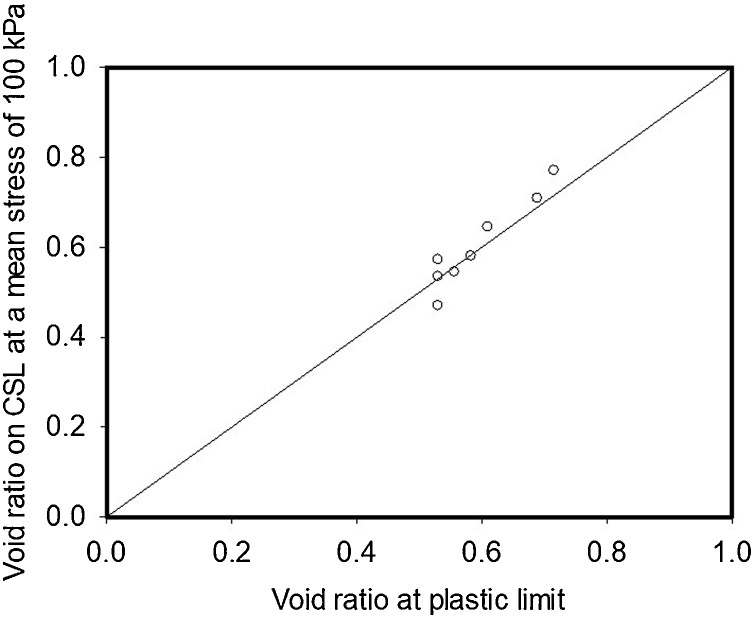
The void ratio for the soil in the critical state at an effective stress of 100 kPa, obtained by interpolation with the curves shown in Fig. 2, Fig. 3, plotted against the void ratio of soil at the plastic limit in the thread rolling experiment, The 1:1 line is also shown.

The plastic limit test, based on hand rolling threads of soil, reduces soil structure to the critical state, where structure is only textural. Although the exact effective stress within the rolled thread is not known, the void ratio at the plastic limit is highly correlated with the void ratio on the critical state line at a mean effective stress of 100 kPa, obtained by interpolation from the fitted curves, and close to a 1:1 relationship (Fig. 7). The choice of an effective stress of 100 kPa is purely arbitrary. However, it has little effect on the strength of the correlation which accounts for 90 percent of the variation in the critical-state void ratio. Our data (Fig. 7), suggests the plastic limit test might be a useful approach for comparing the density between different soils or treatments that overcomes the effect of any temporal patterns.

## 5 Conclusions

We have reported evidence to show that the compression index is independent of soil organic matter, when the effective stress is used to plot the compression characteristic. By comparison with uniaxial compression data, from the literature, the apparent influence of soil organic carbon on the compression index is more likely to be due to its effect on soil hydraulic properties rather than any intrinsic effects on strength. Our data suggests that the effects of soil organic carbon on strength do not depend on scale. Void ratio at any particular effective stress is strongly correlated with organic carbon content. The plastic limit test appears to be a useful and simple test to allow direct comparison of soil physical behaviour.

## Declaration of Competing Interest

There are no conflicts of interest to disclose.
